# An Investigation of the Mechanics and Sticking Region of a One-Repetition Maximum Close-Grip Bench Press versus the Traditional Bench Press

**DOI:** 10.3390/sports5030046

**Published:** 2017-06-24

**Authors:** Robert G. Lockie, Samuel J. Callaghan, Matthew R. Moreno, Fabrice G. Risso, Tricia M. Liu, Alyssa A. Stage, Samantha A. Birmingham-Babauta, John J. Stokes, Dominic V. Giuliano, Adrina Lazar, DeShaun L. Davis, Ashley J. Orjalo

**Affiliations:** 1 Department of Kinesiology, California State University, Fullerton, CA 92831, USA; moreno.matthewr@csu.fullerton.edu (M.R.M.); fabricerisso@csu.fullerton.edu (F.G.R.); deshaunl.davis@csu.fullerton.edu (D.L.D.); ashley.orjalo@csu.fullerton.edu (A.J.O.); 2 Centre for Exercise and Sport Science, School of Exercise and Health Sciences, Edith Cowan University, Joondalup 6027, Australia; scallag5@our.ecu.edu.au; 3 Department of Kinesiology, California State University, Northridge, CA 91330, USA; tricia.tomita.228@my.csun.edu (T.M.L.); alyssa.stage.634@my.csun.edu (A.A.S.); samantha.birminghambabauta.162@my.csun.edu (S.A.B.-B.); john.stokes.91@my.csun.edu (J.J.S.); dominic.giuliano.871@my.csun.edu (D.V.G.); adrina.lazar.957@my.csun.edu (A.L.)

**Keywords:** bar velocity, peak power, mean force, upper-body strength, sticking point, linear position transducer

## Abstract

The close-grip bench press (CGBP) is a variation of the traditional bench press (TBP) that uses a narrower grip (~95% of biacromial distance (BAD)) and has potential application for athletes performing explosive arm actions from positions where the hands are held close to the torso. Limited research has investigated CGBP mechanics compared to the TBP. Twenty-seven resistance-trained individuals completed a one-repetition maximum TBP and CGBP. The TBP was performed with the preferred grip; the CGBP with a grip width of 95% BAD. A linear position transducer measured lift distance and duration; peak and mean power, velocity, and force; distance and time when peak power occurred; and work. Pre-sticking region (PrSR), sticking region, and post-sticking region distance and duration for each lift was measured. A repeated measures ANOVA was used to derive differences between TBP and CGBP mechanics (*p* < 0.01); effect sizes (*d*) were also calculated. A greater load was lifted in the TBP, thus mean force was greater (*d* = 0.16–0.17). Peak power and velocity were higher in the CGBP, which had a longer PrSR distance (*d* = 0.49–1.32). The CGBP could emphasize power for athletes that initiate explosive upper-body actions with the hands positioned close to the torso.

## 1. Introduction

The bench press is one of the foundation exercises used to develop upper-body pushing strength [1,2,3]. This exercise is customarily performed from a supine position on a bench, with a barbell held above the chest with extended arms. The bar is then lowered to the chest such that it contacts just above the xiphoid process, before the bar is forcefully pressed upwards until the elbows are extended [1,2]. The traditional bench press (TBP) is performed with a barbell, however, variations include the bench press being performed with a Smith machine or with dumbbells [4]. The grip width adopted in the TBP with a barbell is usually a preferred width where the individual feels they can lift the greatest load. This will generally mean that the hands are positioned on the bar in a position that is wider than the shoulders, such that when the bar contacts the chest, the elbows will form an approximate 90° angle at the bottom position [5]. The preferred TBP grip width should generally fall within a range of 165–200% of biacromial distance (BAD) [6]. However, there are issues associated with the extremes of this grip width range position that could potentially increase the risk of shoulder injury for the individual [7,8,9].

Green and Comfort [9] noted that the TBP, especially one that features a hand spacing of ≥200% BAD, can place the shoulder joint in a position approaching 90° of abduction. This has been described as an ‘at-risk’ position for shoulder joint injuries [10]. The TBP can be manipulated by adjusting the grip width, which should alter the stress placed on the shoulders [8]. For example, using a grip width of ≥150% BAD has been said to decrease the risk of injury from the bench press exercise [7,8]. What should also be noted is that changes to grip width in the bench press will also change the biomechanics and muscle activation patterns of the lift [1,3,5,6]. Coaches should be conscious of these changes, whether they are adjusting the bench press to reduce excessive abduction of the shoulders, or attempting to induce a sport-specific adaptation.

One variation of the TBP that could be used for sport-specific adaptations is the close-grip bench press (CGBP). The CGBP is typically performed to place greater emphasis on the triceps brachii versus other prime movers such as pectoralis major or deltoids [3,11], as there is less shoulder abduction during this exercise which keeps the hands closer to the torso [1]. Adaptations from this exercise also have the potential for crossover into athletic performance. There are many upper-body pushing actions required in sports where the arms are positioned close to the frame of the torso prior to an extension of the elbows. Some examples include fending in rugby union or league [12,13], blocking in American football [14], or performing a chest pass in basketball and netball [15,16]. The CGBP has been performed with a grip width of 95% BAD within the literature [6], and this places the hands in a position that could relate to these sport-specific actions. However, there has been relatively little analysis of the CGBP, especially with a grip width that places the hands within the torso frame. This means that the arms are adducted at the shoulders and extended at the elbows to keep the hands relatively level with the chest. If strength and conditioning coaches wish to program an exercise such as the CGBP for their athletes, they should have information as to what the resultant power, velocity, force, and work may be for this type of exercise. There is currently no information that illustrates this in the scientific literature.

Another important component of the CGBP that has not been investigated in great detail is the sticking region (SR). The SR has been defined as the section of the lift where the upward velocity of the bar is momentarily decreased [17], and has been recognized as the period from peak barbell velocity until the first local minimum velocity [18,19]. The first local minimum velocity occurs when the upward barbell movement decelerates or even stops completely for a short time during the lift [19]. Several studies that have investigated the bench press have located the SR using this operational definition [1,4,18,19]. Understanding the SR of maximal lifts is important for strength and conditioning coaches for the avoidance of injury and continued progress in strength adaptations for their athletes [20]. Gomo and Van Den Tillaar [1] found that the peak velocity, and local minimum velocity, occurred at a higher position above the chest for a maximal bench press performed with a narrow grip (0.39 ± 0.04 m) by male powerlifters. However, no research has documented whether a grip width of 95% BAD in a CGBP will lead to changes in the SR for this exercise compared to the TBP. In addition to this, the relative distance (bar height as a percentage of lift distance or bar displacement) of the SR for a maximal close-grip bench press has not been described, nor has the relative time.

Therefore, this study investigated the mechanics and SR of the CGBP versus that of the TBP during a one-repetition maximum (1RM) lift. The TBP was performed with the preferred grip width for the individual. The CGBP was performed with a grip width of 95% BAD [6]. Resistance-trained individuals performed 1RM lifts for both the TBP and CGBP, and each lift was measured with a linear position transducer. The data that was recorded for this study included: lift distance (i.e., bar displacement) and duration; peak and mean power, velocity, and force; the timing and location of peak power; work; and the distance and duration of the pre-sticking region (PrSR), SR, and post-sticking region (PoSR), relative to lift distance and duration, respectively. The use of a linear position transducer to measure each lift was conducted to ensure the data would have practical value to the strength and conditioning coach, due to the use of this equipment within the field [21,22,23,24,25]. It was hypothesized that the CGBP would have a longer lift distance and lift time compared to the TBP, and this would lead to a lower 1RM and force. However, power and velocity would be higher because the load will be lighter in the CGBP [1]. Furthermore, it was hypothesized that the SR in the CGBP would be longer, both in distance and duration, when compared to the TBP.

## 2. Materials and Methods

### 2.1. Subjects

Twenty-seven resistance-trained individuals (age = 23.7 ± 3.9 years; height = 1.72 ± 0.09 m; body mass = 77.5 ± 16.5 kg), including 21 males (age = 24.1 ± 4.3 years; height = 1.75 ± 0.06 m; body mass = 81.8 ± 15.8 kg) and 6 females (age = 22.7 ± 1.4 years; height = 1.58 ± 0.06 m; body mass = 62.3 ± 7.4 kg), volunteered to participate in this study. Subjects were recruited from the student population at the university. Data was combined for males and females, which has been done in previous maximal strength research [25,26,27,28]. Preliminary analysis of the TBP and CGBP also indicated similar patterns of lift mechanics for each exercise between genders. All subjects were required: to be currently resistance training (≥three hours per week) with a focus on either hypertrophy or maximal strength development; have a resistance training history (≥two times per week) of at least two years, and be experienced with completing maximal lifts; be experienced with the TBP and CGBP; and free from any musculoskeletal disorders that would influence their ability to complete the study. G*Power software (v3.1.9.2, Universität Kiel, Kiel, Germany) was used post hoc to confirm that the sample size of 27 was sufficient for a repeated measures analysis of variance (ANOVA), within factors analysis, and ensured the data could be interpreted with a small effect level of 0.10 [29], and a power of 0.80 when significance was set at 0.01 [30]. The institutional ethics committee approved the procedures used in this study. All subjects received a clear explanation of the study, including the risks and benefits of participation, and written informed consent was obtained prior to testing.

### 2.2. Procedures

Subjects completed one testing session, and all assessments were conducted in the teaching gym at the university. Prior to data collection, the subject’s age, height, body mass, and BAD were recorded. Height was measured barefoot using a portable stadiometer (seca, Hamburg, Germany), while body mass was recorded by electronic digital scales (Tanita Corporation, Tokyo, Japan). BAD was measured as the distance between the acromion processes for each shoulder. These landmarks were palpated and marked with a makeup pencil, and then measured with a handheld tape measure (Lufkin, Sparks, MD, USA). The measurement was taken as a straight line between these landmarks, such that the musculature of the upper back and shoulders did not influence the distance. The 1RM for the TBP and CGBP were both assessed within the one session, the procedures of which will be described. The exercise that was completed first was randomized amongst the sample via the randomization function in a Microsoft Excel spreadsheet (Microsoft Corporation, Redmond, DC, USA). Subjects refrained from intensive upper-body exercise and maintained a standardized dietary intake in the 24-h period prior to testing, and were permitted to consume water as required throughout the testing session. No bench press suits, weightlifting belts, or other supportive garments were permitted. 

### 2.3. 1RM TBP and CGBP Strength Testing

The 1RM was measured for both the TBP and CGBP, and as stated the testing order for these lifts was randomized amongst the sample. A standard Olympic bar and weight plates (American Barbell, San Diego, CA, USA) were used for both tests, which were conducted within a power rack (American Barbell, San Diego, CA, USA). Free weights were used in this study as they have greater application in the training of strength-trained individuals as muscle activation is increased [31], and greater loads can be lifted when compared to a guided bench press performed on equipment such as a Smith machine [32]. Indeed, Cotterman et al. [32] noted that the Smith machine is actually restrictive on the required movement patterns for a maximal TBP. The methods here will describe the process if the 1RM for the TBP was completed first, and the procedures for the lifts were adapted from the literature [1,2,33,34,35]. When performing the TBP, subjects laid supine on a flat bench (American Barbell, San Diego, CA) with their feet flat on the floor, and their head, shoulders, and buttocks flat to the bench. Subjects self-selected the hand position for the 1RM TBP [1,36,37,38,39], and used a pronated grip. Following instructions used by Young et al. [36], subjects were told to select their ‘strongest position’ for the grip width in the TBP. The distance between the index fingers on the bar was measured, such that this could be made relative to the subject’s BAD. The distance was marked on the Olympic barbell with thin strips of athletic tape to ensure subjects placed their hands on the bar at the same position for each repetition. The subject unracked the bar with assistance from a spotter if required, and began the lift with the arms extended and elbows locked [1,2]. The ‘touch-and-go’ procedure was adopted, in that the bar was required to touch the chest before being pressed to full arm extension [33]. A repetition was deemed to be successful when the bar was moved from the chest to a position of full elbow extension [34]. Failure to do this, or bouncing the bar on the chest, disqualified a repetition. A spotter was positioned behind the bar for assistance with lift-off if required and for safety, but did not touch the bar except in the event of a failed lift [5]. To warm-up for the 1RM, procedures were adapted from Stock et al. [35], and rest periods of two minutes were provided between sets. Subjects initially completed 8–10 repetitions at 50% of their estimated 1RM, followed by 3–5 repetitions at 85% of the estimated 1RM. Subjects then completed a single repetition with 90% of the estimated 1RM, before attempting their first 1RM attempt. If the subject was successful, loads would increase by increments of 2.5 kg until the subject failed to complete a repetition. No more than five attempts were needed before the 1RM was reached. Three minutes of rest was provided between the 1RM efforts.

After a 10-min recovery period, subjects then completed the CGBP. The body position and parameters that determined a successful lift were the same as that for the TBP, except for the difference in grip width. The grip width used was 95% of BAD [6], as this placed the hands in a position inside the shoulders. This hand position is similar to that required in upper-body pushing motions in sports [14,15,16], and thus was adopted in the current study. As for the TBP, the grip width was marked on the barbell with athletic tape, and a pronated grip was used. Following the methods of Gomo and Van Den Tillaar [1], the warm-up for the second strength test began by completing 3–5 repetitions at 85% of the subjects’ estimated 1RM, and then one repetition with 90% 1RM. Subjects then attempted their first 1RM attempt following a 3-min recovery period, and this process continued until the 1RM was attained. For both the TBP and CGBP, absolute strength was taken as the maximum load lifted for one repetition. The 1RM was also scaled relative to body mass for both lifts according to the formula: relative 1RM (kg·BM^−1^) = 1RM∙body mass^−1^.

Data was recorded during each TBP and CGBP 1RM attempt by a GymAware Powertool linear position transducer (Kinetic Performance Technology, Canberra, Australia). This equipment was used in this study as it is more practical for the strength and conditioning coach to measure lifting data in this manner [21,22,23,24,25], as opposed to using motion capture. The GymAware Powertool features a spring-loaded retractable cable that passes around a spool integrated with an optical encoder [40]. The external end of the cable was attached on the inside of the barbell (i.e., inside the plates, and on the outer part of the grip section of the bar) for both the TBP and CGBP, and provided no additional resistance to the bar. The unit was then placed on the floor directly underneath the bar, with the magnetic bottom positioned on top of a weight plate to ensure it did not move during each lift. The linear position transducer recorded velocity and the movement of the bar at 50 Hertz (Hz); barbell load was entered into the software to calculate power and force for every 3 millimeters of bar movement [40]. Data for each 1RM attempt was collected and stored on an iPad handheld device (Apple Inc., Cupertino, CA, USA) before being uploaded to an online database. The data was then exported from this database and entered into Microsoft Excel (Microsoft Corporation^TM^, Redmond, WA, USA) prior to statistical analyses.

Several variables were measured for the TBP and CGBP, and only concentric variables were considered for this study. These included: lift distance (i.e., displacement of the bar from lift initiation to lockout) and duration in seconds (s); peak and mean power (watts; w) and the actual (measured in m and s, respectively) and relative (both measured as a percentage; %) distance and time when it occurred during the lift; peak and mean velocity (meters per second; m·s^−1^); peak and mean force (Newtons; N); and work (joules; J). Power and force variables were derived relative to the load on the bar, which was entered into the GymAware software. The GymAware Powertool has been shown to produce reliable and valid data [22,41,42]. Furthermore, Lockie et al. [25] reviewed the research documenting reliability data for the GymAware Powertool. For instance, Black [41] found typical errors of measurements for distance of 0.00 m, duration of 0.01–0.02 s, and velocity of 0.01 m·s^−1^. Hori and Andrews [42] reported high reliability for peak velocity (coefficient of variation (CV) = 1.1–4.6%), with peak force being slightly less reliable (CV = 4.1–7.9%). Concentric power has been found to have coefficients of variation of 1.0–3.02% across different strength exercises [22]. Similar to Lockie et al. [25], all variables measured in this research were considered to have acceptable reliability.

Procedures were adapted from the literature to determine the SR for the TBP and CGBP [19]. The velocity and displacement curves for each 1RM lift was analyzed within the GymAware software to determine when peak velocity occurred within each lift, and the SR was defined as the period from the first peak barbell velocity until the first local minimum velocity [17,19]. As stated, three periods were assigned to each bench press, which were the:

- PrSR: time from lowest barbell point until maximal barbell velocity.
- SR: time from maximal barbell velocity until first local minimum barbell velocity.
- PoSR: time from the instant vertical acceleration of the barbell became positive again until the completion of the lift.

**Figure 1:**
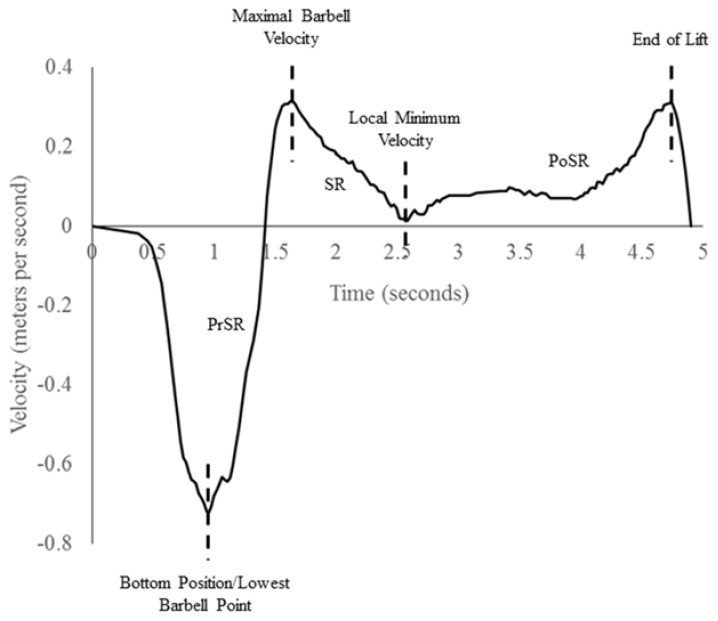
Example velocity curve for a bench press exercise indicating the pre-sticking region (PrSR), sticking region (SR), and post-sticking region (PoSR).

The required landmarks were estimated visually from the velocity curve calculated from the 1RM [25,43,44], and are shown in Figure 1. The distance and duration of each of these regions were expressed as a percentage relative to lift distance and duration, respectively.

### 2.4. Statistical Analysis

Data analysis procedures were adapted from Lockie et al. [25]. All statistics were computed using the Statistics Package for Social Sciences Version 24.0 (IBM, Armonk, NY, USA). Descriptive statistics (mean ± standard deviation (SD); 95% confidence intervals (CI)) were used to provide the profile for each measured parameter. Several statistical approaches were used in this study. Stem-and-leaf plots were used to ascertain whether there were any outliers in the data for each variable, and any outliers were treated via a winsorization method [45,46,47]. Similar to Lockie et al. [25], a repeated measures ANOVA was used to compare differences in the bench press variables, with significance set at *p* < 0.01. Although *p* < 0.05 is often used as the significance level in sport science research, the more stringent significance level was used due to the number of variables analyzed in this study [25,48]. The within-subjects measure (i.e., which bench press was completed) represented the TBP and CGBP conditions. As only two repeated measures were employed, the assumption of sphericity, determined by Mauchly’s test of sphericity, was not applicable. All other repeated measures ANOVA assumptions were considered. Effect sizes (*d*) were also calculated for the between-lift comparison, where the difference between the means was divided by the pooled SD [49]. The formula used was: (Mean_1_ − Mean_2_) ÷ ((SD_1_ + SD_2_) ÷ 2)^−1/2^. A *d* less than 0.2 was considered a trivial effect; 0.2 to 0.6 a small effect; 0.6 to 1.2 a moderate effect; 1.2 to 2.0 a large effect; 2.0 to 4.0 a very large effect; and 4.0 and above an extremely large effect [29]. Additionally, 95% CI were derived for the *d* calculations.

## 3. Results

The data for grip width, 1RM and relative strength values, and lift distance and duration are shown in Table 1. There was a very large, significant difference in the grip width adopted between the TBP and CGBP, which was to be expected. The mean preferred grip width adopted by subjects in this study was equivalent to 175% BAD. A 5% significantly greater load was lifted in the TBP compared to the CGBP, although the effect was trivial. Subjects recorded a 6% greater relative strength measure for the TBP compared to the CGBP, and this effect was small. There were no significant differences in lift distance or duration between the TBP and CGBP.

The power, velocity, and force data is displayed in Table 2. Peak power was 20% greater in the CGBP, although the effect was small. Peak power also occurred at a significantly further distance away from the bottom position of the lift for the CGBP. However, the relative percentage of lift distance when peak power occurred was not significantly different between the TBP and CGBP, nor was the timing of peak power. Mean power was not significantly different between the TBP and CGBP. A 23% greater peak velocity was achieved in the CGBP, which had a large effect. There were no significant differences between the two 1RM bench press exercises for mean velocity, peak force, and work. Mean force was 5% greater for the TBP, although the effect was trivial.

**Figure 2:**
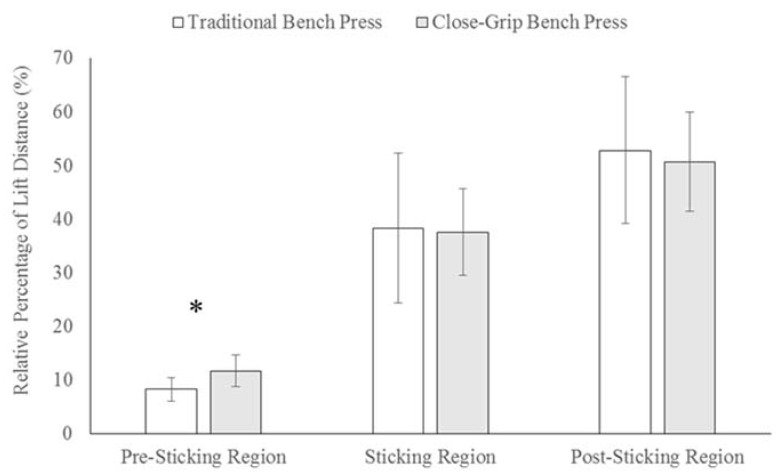
Distances of the pre-sticking region, sticking region, and post-sticking region relative to lift distance in a maximal traditional and close-grip bench press in resistance-trained men and women (*n* = 27); * Significant (*p* < 0.01) difference between the CGBP and the TBP.

**Figure 3:**
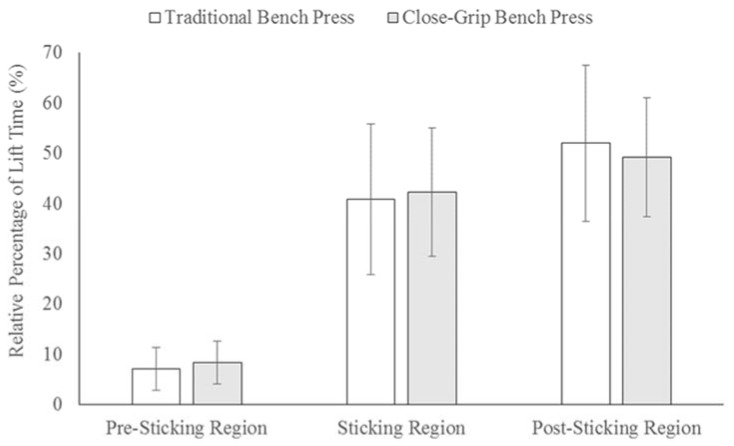
Durations of the pre-sticking region, sticking region, and post-sticking region relative to lift duration in a maximal traditional and close-grip bench press in resistance-trained men and women (*n* = 27).

Figure 2 displays the distances for the three regions in the TBP and CGBP. There was a significantly (*p* < 0.001) greater relative PrSR distance for the CGBP compared to the TBP, which had a large effect (*d* = 1.32). There were no significant differences between the bench press exercises for the SR (*p* = 0.805; *d* = 0.06) or PoSR (*p* = 0.440; *d* = 0.19) distances. As shown in Figure 3, there were no significant differences in the relative duration of the PrSR (*p* = 0.137; *d* = 0.31), SR (*p* = 0.636; *d* = 0.10), or PoSR (*p* = 0.372; *d* = 0.20) between the TBP and CGBP.

## 4. Discussion

This is the first study to provide a detailed analysis of the mechanics of the CGBP as measured by a linear position transducer compared to the TBP in resistance-trained men and women. The results indicated that in addition to a greater load being lifted in the TBP, there were select mechanical differences between this exercise and the CGBP. A higher peak power and velocity were achieved in the 1RM CGBP, which would likely relate to the lighter load lifted. The PrSR was longer in the CGBP, but the SR and PoSR distance did not vary between the two bench press exercises. Given these results, there could be some advantages to programming the CGBP regularly in an athlete’s training regimen, especially for those athletes (e.g., athletes from contact sports such as rugby and American football, and court sports such as basketball and netball) who perform actions similar to that required in the CGBP during match-play.

It was hypothesized that a greater load would be lifted in the TBP, and this was the case in this study. Gomo and Van Den Tillaar [1] found that a TBP performed with both a wide (0.75 ± 0.10 m; 131.5 ± 22.9 kg) and medium grip width (0.57 ± 0.06 m; 126.5 ± 21.6 kg) resulted in a significantly greater load lifted when compared to a narrow grip width (0.39 ± 0.04 m; 122.1 ± 19.4 kg) in male powerlifters. Wagner et al. [6] also found resistance-trained male college students generated less force in a maximal bench press performed with a grip width of 95% BAD (1098.19 ± 137.10 N) when compared to grips widths of 165% (1165.87 ± 154.66 N) and 200% (1176.06 ± 165.15 N) BAD. The heavier load lifted in the TBP would likely relate to a superior mechanical advantage gained from the preferred grip width position adopted in this lift [1], as well as greater activation of the pectoralis major [3,5,11]. The mean grip width adopted by subjects in this study (175% BAD) was below that considered to place the shoulders at risk of injury (i.e., ≥200% BAD) [9]. Furthermore, this grip width falls within the range of 165–200% BAD found by Wagner et al. [6] where the strongest grip widths occurred for male college students.

There was a significant difference between the 1RM loads for the TBP and CGBP in this research. However, lift distance and duration were not significantly different between the TBP and CGBP. This was counter to the study’s hypothesis, as it was expected that the grip position for the CGBP would result in further bar displacement as there is less shoulder abduction and greater shoulder flexion [1]. However, any technical changes may have influenced the resulting bar path, which could have affected the resultant lift distance and duration for the CGBP compared to the TBP [6]. Limited differences in lift times for bench press variations have been previously documented in the literature. When considering a 1RM bench press completed with a barbell, on a Smith machine, and with dumbbells by resistance-trained males, van den Tillaar and Saeterbakken [4] found no significant differences between lift times. Future research could investigate the CGBP performed with a grip width of 95% BAD using three-dimensional motion capture for a more detailed analysis of technique and bar path. Nonetheless, the results from the study indicated that although the 1RM loads were different, there were minimal differences between the lift distance and duration of the 1RM TBP and CGBP when measured by a linear position transducer.

Greater peak power and peak velocity was achieved in the CGBP compared to the TBP. Gomo and Van Den Tillaar [1] also found that male powerlifters generated a higher bar velocity with a 1RM bench press performed with a narrow grip compared to lifts with wider hand positions. This would relate to the lighter 1RM load lifted for the CGBP both in the current study, and in the research conducted by Gomo and Van Den Tillaar [1]. These results have clear application for training in the athletic field. As noted earlier, fending in rugby [12,13], blocking in American football [14], or performing a chest pass in basketball and netball [15,16] are all explosive upper-body actions. In order to perform these actions effectively on the field or court, the athlete must extend the elbows with high velocity and power. The CGBP could be used as an exercise to develop the underlying strength capacity specific to these movements. In addition to this, those individuals who need to use a narrower grip due to shoulder issues [7,8] can do so with the knowledge that they could still experience positive adaptations with regards to power and speed of movement. Although future research is required to confirm any potential training effects from the long-term use of the CGBP, this exercise could be programmed to emphasize sport-specific upper-body power in athletes.

Greater mean force was generated in the TBP when compared to the CGBP, which supports the findings from Wagner et al. [6]. These results were also expected, given that a greater 1RM load was lifted in the 1RM TBP compared to the CGBP. Indeed, mean force was likely greater because more force was needed throughout the concentric phase of the lift in order to move the heavier load. Furthermore, a TBP performed with the preferred grip should optimize the moment arms at the shoulder and elbow specific to the individual [1]. This should result in greater torque generation at these joints, which in turn should increase the force that can be generated against the bar. However, there were no significant differences in the work produced between the work done in the TBP and CGBP. As work equates to force multiplied by distance, the ratio between these two variables was conceivably similar for the TBP and CGBP, even if the actual force and distance data were different. This also has implications for individuals who select a narrower grip for the bench press. During maximal lifting, it could be expected that even if the mean force is lower due to a lighter load, the work completed in a CGBP would still be comparable to the TBP.

A SR was clearly identified for the TBP and CGBP for all subjects, which was in agreement with the literature [1,4,18,39]. There were no significant differences between the PrSR, SR, and PoSR durations relative to lift time in TBP and CGBP, nor were there differences in the SR and PoSR distance. However, there was a significant difference, with a large effect, between the PrSR duration in the TBP and CGBP. The CGBP had a longer PrSR, which would have also influenced when peak power occurred during this lift, which was a significantly further distance away from the bottom position when compared to the TBP. Gomo and Van Den Tillaar [1] also found that a bench press performed with a narrow grip resulted in peak velocity occurring further away from the chest when compared with medium- and wide-grip bench presses in male powerlifters. The longer PrSR distance for the CGBP could relate to the mechanics of the lift, which as stated, features more shoulder flexion and less shoulder abduction [1]. As this reduces the moment arms for the shoulder and elbow [1], the work done during the PrSR may be relatively more important for the CGBP in order to move the bar. Indeed, as the PrSR immediately precedes the SR, it can add to force generation capabilities through the difficult region of the lift [22,50]. What these results highlight is that the SR for the maximal CGBP will start further away from the chest when compared to the TBP. 

There were several limitations for this study that should be acknowledged. Data for men and women were combined to increase the statistical power of this study. Even though this approach has been used in previous research investigating strength exercises [25,26,27,28], this could be problematic given the established strength differences that generally exist between men and women [51,52]. Future studies should analyze whether there are gender-specific responses to a 1RM CGBP. A non-guided free weight Olympic bar was used for both the TBP and CGBP, which may have influenced the vertical displacement achieved for both lifts. However, this equipment was used due to greater practical application, especially for strength-trained individuals [31,32]. In addition to this, although the subjects were resistance-trained, they may not necessarily have been ‘strong’. For example, the 1RM for the TBP for the males and females in this study was 87.35 ± 27.23 kg; the male powerlifters in the study conducted by Gomo and Van Den Tillaar [1] had a wide-grip bench press of 131.5 ± 22.9 kg. Future research could investigate the mechanics of the CGBP in stronger populations, such as elite athletes involved in pushing sports (e.g., American football and rugby), and male and female powerlifters. As previously stated, further investigations of the CGBP could utilize motion capture to provide a more detailed analysis of the bar path and movement kinematics. Indeed, bar mechanics was measured by a linear position transducer recording data at 50 Hz, which is lower than most motion capture systems. Nonetheless, the linear position transducer used in this study records reliable and valid data [22,41,42], and was adopted because of its practical application, ease of use in the field, and provision of useful information for the strength and conditioning coach [21,22,23,24,25].

## 5. Conclusions

This study found that when compared to the TBP, a 1RM CGBP resulted in a lighter maximal load, greater peak power and velocity, lower mean force, and greater PrSR distance in resistance-trained men and women. Thus, strength and conditioning coaches could program the CGBP for sport-specific adaptations in explosive arm actions. This could be of particular value for those athletes that need to initiate this explosive elbow extension from a position that begins with the hands held close to the frame of the torso. Furthermore, individuals who need to use a narrower grip due to shoulder issues could still experience positive adaptations from the CGBP, due to the higher power generated, and relative similarities in work performed when compared to the TBP. The PrSR will have a longer distance in the CGBP, so individuals who either complete or spot the CGBP should be cognizant of this technical difference when compared to the TBP. Lastly, it must be stated that the results from this study do not suggest that the TBP should be eliminated from the training regimes of athletes. Rather, the CGBP could be used for variation when programming upper-body pushing strength exercises for athletes.

## Figures and Tables

**Table 1:** Descriptive statistics (mean ± standard deviation (SD); 95% Confidence Intervals (CI)) for absolute and relative strength, lift distance, and lift duration for the one-repetition maximum (1RM) traditional bench press (TBP) and close-grip bench press (CGBP) in resistance-trained individuals (*n* = 27).

Variable	TBP	CGBP	*p* Value	*d*	*d* Strength
Grip Width (m)	0.60 ± 0.11	0.34 ± 0.04 *	<0.001	3.14	Very Large
	(0.56–0.65)	(0.32–0.35)		(2.31–3.89)	
1RM Load (kg)	87.35 ± 27.23	83.03 ± 24.67 *	<0.001	0.17	Trivial
	(75.12–96.93)	(73.07–93.00)		(−0.37–0.70)	
Relative Strength (kg·BM^−1^)	1.12 ± 0.27	1.06 ± 0.24 *	<0.001	0.23	Small
	(1.00–1.21)	(0.97–1.16)		(−0.30–0.77)	
Lift Distance (m)	0.41 ± 0.04	0.43 ± 0.05	0.018	0.44	Small
	(0.40–0.43)	(0.41–0.45)		(−0.10–0.98)	
Lift Duration (s)	3.47 ± 1.43	2.98 ± 1.03	0.044	0.39	Small
	(2.91–4.02)	(2.56–3.39)		(−0.15–0.93)	

kg: kilograms; kg·BM^−1^: kilograms lifted per kilogram body mass; * Significantly (*p* < 0.01) different.

**Table 2:** Descriptive statistics (mean ± SD; 95% CI) for peak (PP) and mean (MP) power, time at when peak power occurred in the lift, peak (PV) and mean (MV) velocity, time at when peak velocity occurred in the lift, peak and mean force, and work characteristics for the one-repetition maximum traditional bench press (TBP) and close-grip bench press (CGBP) in resistance-trained individuals (*n* = 27).

Variable	TBP	CGBP	*p* Value	*d*	*d* Strength
PP (w)	313.18 ± 105.94	376.48 ± 149.66 *	0.001	0.49	Small
	(265.26–350.36)	(316.03–436.93)		(−0.06–1.02)	
PP Distance (m)	0.024 ± 0.010	0.035 ± 0.016 *	0.006	0.82	Moderate
	(0.021–0.029)	(0.028–0.041)		(0.26–1.37)	
PP Distance (%)	6.28 ± 3.02	8.15 ± 3.99	0.068	0.53	Small
	(5.22–7.66)	(6.53–9.76)		(−0.02–1.06)	
Time at PP (s)	0.17 ± 0.06	0.17 ± 0.05	0.784	<0.01	Trivial
	(0.15–0.20)	(0.15–0.20)		(−0.53–0.53)	
Time at PP (%)	6.16 ± 3.90	6.74 ± 3.34	0.412	0.16	Trivial
	(4.69–7.72)	(5.40–8.09)		(−0.38–0.69)	
MP (w)	168.71 ± 70.33	190.81 ± 87.82	0.088	0.28	Small
	(136.96–193.41)	(155.34–226.28)		(−0.26–0.81)	
PV (m·s^−1^)	0.35 ± 0.06	0.43 ± 0.07 *	<0.001	1.23	Large
	(0.32–0.37)	(0.40–0.46)		(0.63–1.79)	
MV (m·s^−1^)	0.20 ± 0.06	0.23 ± 0.07	0.023	0.46	Small
	(0.17–0.22)	(0.20–0.26)		(−0.09–0.99)	
Peak Force (N)	1124.24 ± 378.37	1107.39 ± 394.63	0.579	0.04	Trivial
	(949.75–1256.10)	(948.00–1266.79)		(−0.49–0.58)	
Mean Force (N)	861.74 ± 269.48	820.03 ± 240.36 *	<0.001	0.16	Trivial
	(740.72–956.59)	(722.95–917.12)		(−0.37–0.70)	
Work (J)	265.64 ± 150.31	239.87 ± 128.94	0.288	0.18	Trivial
	(204.93–326.36)	(188.86–290.88)		(−0.35–0.72)	

w: watts; %: percent; m·s^−1^: meters per second; N: Newtons; J: joules; * Significantly (*p* < 0.01) different from the TBP.

## Abbreviations

The following abbreviations are used in this manuscript:

TBP	Traditional bench press
BAD	Biacromial distance
CGBP	Close-grip bench press
SR	Sticking region
m	Meters
1RM	One-repetition maximum
PrSR	Pre-sticking region
PoSR	Post-sticking region
kg	Kilograms
ANOVA	Analysis of variance
BM	Body mass
s	Seconds
w	Watts
m·s^−1^	Meters per second
N	Newtons
J	Joules
CV	Coefficient of variation
SD	Standard deviation
CI	Confidence intervals
*p*	Significance
*d*	Effect size
PP	Peak power
MP	Mean power
PV	Peak velocity
MV	Mean velocity
